# Alcohol use disorder symptoms are associated with greater relative value ascribed to alcohol, but not greater discounting of costs imposed on alcohol

**DOI:** 10.1007/s00213-018-4922-8

**Published:** 2018-05-09

**Authors:** Lee Hogarth, Lorna Hardy

**Affiliations:** School of Psychology, University of Exeter, Washington Singer Building, Perry Road, Exeter, EX4 4QG UK

**Keywords:** Alcohol use disorder symptoms, Relative value, Cost discounting, Concurrent choice

## Abstract

**Rationale:**

Alcohol dependence is characterised by persistent drinking despite health, social and economic costs. Behavioural economics has proposed two explanations for the persistence of alcohol use despite costs. Dependent individuals may (a) ascribe excessively high value to alcohol, such that costs associated with alcohol are exceeded, and/or (b) they may discount (neglect) the costs associated with alcohol.

**Methods:**

To test these predictions, the current study recruited 127 student drinkers who reported varied alcohol use disorder symptom severity in the Alcohol Use Disorders Inventory Test (AUDIT; mean = 11.17, 69% above the hazardous cutoff). Participants made concurrent forced choices between alcohol and food points under conditions that manipulated the magnitude of points (1, 2 or 3) and the delay to receive points (0 or 3 s). Alcohol value was indexed by preferential choice of alcohol versus food points, whereas sensitivity to costs was indexed by the decrease in alcohol choice when food points were of greater magnitude (sensitivity to opportunity costs) and when alcohol points were delayed (sensitivity to delay costs).

**Results:**

Percent choice of alcohol over food varied consistently with the relative magnitude of reward points offered (*p* < .001) and with time delays imposed on reward points (*p* < .001). AUDIT scores were associated with greater alcohol versus food choice across all conditions (*p* = .001). As alcohol use disorder symptom severity increased, the sensitivity of alcohol choice to the relative magnitude of points (*p* = .29) and time delays (*p* = .62) remained unchanged, suggesting no differential discounting of opportunity or delay costs imposed on alcohol. In contrasts of AUDIT categories, there was comparable sensitivity to costs across groups defined as low-risk (*N* = 39), hazardous (*n* = 57), harmful (*n* = 20) and possible dependent drinkers (*n* = 11).

**Conclusions:**

Alcohol use disorder symptom severity is associated with greater relative value ascribed to alcohol, but not with greater discounting of opportunity or delay costs imposed on alcohol. Despite limitations of the current study, it may be concluded that cost discounting plays a lesser role in dependence than previously thought.

## Introduction

A key diagnostic feature of alcohol dependence is that dependent individuals will continue to drink even when doing so brings about negative health, social and economic consequences (American Psychiatric Association 2013). Behavioural economic theory has proposed two explanations for continued drinking in the face of rising costs in dependent individuals. First, more dependent drinkers may ascribe excessively high value to alcohol, such that costs associated with alcohol are exceeded, so drinking persists despite costs (MacKillop 2016). The second possibility is that more dependent drinkers discount (i.e. neglect) the costs associated with drinking in their decision-making, such that drinking persists despite costs (Belin et al. 2008; Bickel et al. 2014; Mitchell 2003). It is important to distinguish these two possibilities to clarify the psychological mechanism(s) underpinning dependence. The purpose of the current study was to test, using a novel concurrent choice procedure, whether alcohol use disorder symptom severity in student drinkers would be associated with greater relative value ascribed to alcohol and/or greater discounting of costs imposed on alcohol.

Evidence that alcohol dependence is associated with greater value ascribed of alcohol comes from human demand tasks. In these tasks, drinkers report the amount of alcohol they would hypothetically consume across increasing prices. The intensity of demand (maximum consumption at zero or low cost) is considered to be a relatively pure index of the value of alcohol unaffected by sensitivity to costs, whereas peak expenditure (or Omax) and elasticity may reflect both alcohol value and cost sensitivity. Intensity of demand for alcohol correlates with various proxies for dependence, including drinks consumed per week (MacKillop and Murphy 2007), episodes of heavy drinking per week (Murphy and MacKillop 2006) and alcohol-related problems (Murphy et al. 2009). Similarly, in concurrent choice procedures, where drinkers choose between alcohol and food rewards (points or pictures), preference for the alcohol reward is associated with alcohol use disorder symptom severity in both hazardous drinkers recruited from the community (Hardy and Hogarth 2017) and student drinkers (Hardy et al. 2017; Hogarth et al. 2018). These demand and choice data fit with the prediction of economic theory that drinkers with greater dependence symptoms ascribe greater relative value to alcohol, which could underpin persistent drinking despite costs.

In demand tasks, breakpoint—the price at which alcohol consumption drops to zero—is thought to index the extent to which drinkers incorporate price costs into their decision to drink, with higher breakpoints indicating greater cost discounting (MacKillop and Murphy 2007). Evidence is mixed as to whether alcohol dependence is associated with higher breakpoints. Higher breakpoints have been found to be associated with drinking heaviness in students (Murphy and MacKillop 2006), but not with alcohol dependence symptom severity in adults (MacKillop et al. 2010). Importantly, a meta-analysis of this literature found that proxies for alcohol dependence correlated more consistently across studies with measures of intensity than with breakpoint (MacKillop et al. 2015), suggesting that alcohol dependence may be driven by higher value ascribed to alcohol rather than cost discounting. However, one key study found that student drinkers with a family history of alcoholism were less sensitive to the effect of imagined next-day responsibilities on reducing alcohol demand (Murphy et al. 2014) supporting the claim that dependence vulnerability may be linked to discounting costs associated with alcohol.

Another potential source of evidence for cost discounting in alcohol dependence comes from delay discounting tasks. In these tasks, drinkers choose between smaller immediate and larger delayed rewards (alcohol or money). It is typically found that alcohol use disorder symptoms are associated with a greater preference for the smaller immediate reward (Lim et al. 2017; MacKillop et al. 2011; Petry 2001; Vuchinich and Simpson 1998). One interpretation of this result is that dependence is associated with greater sensitivity to time delay costs (not cost discounting), because the value of the reward declines more steeply with delay. However, the typical interpretation is that reduced choice of the delayed reward reflects a restricted temporal horizon, i.e. neglect of future outcomes in decision-making, which arguably includes neglect of future costs associated with drinking (MacKillop et al. 2011). However, this possibility remains to be demonstrated directly. Thus, steeper temporal discounting provides only ambiguous evidence for greater cost discounting as a function of alcohol dependence symptoms.

Deficits in reversal learning can be interpreted as evidence for greater discounting of punishment contingencies in dependent individuals. In the reversal learning task, participants first learn that one response choice has a higher payoff than the alternative choice, before these response-reward contingencies are reversed. Drug users show deficits in reversal learning despite comparable acquisition of the initial contingencies (Ersche et al. 2008; Fortier et al. 2008; Reiter et al. 2016; Vanes et al. 2014). One explanation of these findings is that drug users are less sensitive to punishment of the incorrect choice, enabling persistence of that choice in reversal. However, reversal learning deficits could be due to impaired prediction error coding, cognitive inflexibility or general task disengagement. Furthermore, because the reward and punishment contingencies are confounded in the reversal task, impaired reversal learning cannot be unequivocally attributed to punishment discounting (Ersche et al. 2008).

Perhaps the best evidence that dependence is driven by cost discounting comes from animal studies. Several studies have shown that rats that are impulsive or have been given extended access to the drug (and so are notionally dependence prone) show weaker suppression of drug self-administration by contingent shock punishment, despite comparable baseline self-administration rates to control animals (Belin et al. 2008; Economidou et al. 2009; Pelloux et al. 2007; Pelloux et al. 2015; Vanderschuren and Everitt 2004). These effects suggest that the nominally dependent rats do not ascribe higher value to drugs at baseline, but rather, selectively discount the costs associated with drug self-administration (but see the “Discussion” for counter arguments). The implication is that drug choice in more dependent humans should also be less sensitive to the suppressive effects of costs (i.e. they should discount costs imposed on the drug).

Concurrent choice procedures offer a method for measuring the relative value ascribed to alcohol and sensitivity to costs imposed on alcohol. In concurrent choice procedures, participants choose between a drug reward and a concurrently available natural reward alternative across a series of trials (the two rewards may be points-based, pictures or actually consumed/administered depending on the method). The claim that percent drug choice indexes the relative value ascribed to the drug versus natural reward is supported by the finding that percent drug choice reliably increases with the severity of dependence to alcohol (Hardy and Hogarth 2017; Hardy et al. 2017; Hogarth et al. 2018), cocaine (Moeller et al. 2013; Moeller et al. 2009) and tobacco (Chase et al. 2013; Hogarth and Chase 2011). Importantly, concurrent choice procedures can also index sensitivity to opportunity costs, quantified by the decrease in drug choice that occurs when the magnitude of the competing alternative reward is increased. This measure reflects sensitivity to the cost imposed on the drug choice by the potential loss of the valuable alternative reward (Bickel et al. 1995; Campbell and Carroll 2000; Carroll and Lac 1993; Carroll et al. 1989; Ginsburg and Lamb 2018; Hatsukami et al. 1994; Higgins et al. 1994, 1996; LeSage 2009; Nader and Woolverton 1991, 1992; Stevens Negus 2003). Finally, concurrent choice procedures can index sensitivity to delay costs, quantified by the decrease in drug choice that occurs when a delay is imposed between the choice and receipt of the drug (Ito and Nakamura 1998; Woolverton and Anderson 2006).

The purpose of the current experiment was to test, using a novel concurrent choice procedure, whether alcohol use disorder symptom severity in student drinkers would be associated with greater relative value ascribed to alcohol indexed by greater percent choice of alcohol versus food. Secondly, the study tested whether alcohol choice could be modified by imposing opportunity and delays costs on alcohol, to demonstrate that alcohol choice is an economic decision based on the weighing of rewards and costs. Thirdly, and most importantly, the study tested whether alcohol use disorder symptom severity is associated with greater discounting of opportunity costs imposed on alcohol choice (smaller decrease in alcohol choice when the magnitude of the competing alternative is increased) and greater discounting of delay costs imposed on alcohol choice (smaller decrease in alcohol choice when a delay is imposed on the receipt of alcohol). As far as we are aware, only two experiments have utilised such a method (Vuchinich and Tucker 1983; Vuchinich et al. 1987). In these studies, drinkers completed a concurrent choice procedure for alcohol and money, across conditions where money was manipulated in magnitude and delay. Alcohol choice decreased as the magnitude of the money alternative increased demonstrating the sensitivity of alcohol choice to opportunity costs. Furthermore, alcohol choice increased when a delay was imposed on receipt of the money reward, demonstrating sensitivity to delay costs. However, these studies did not test whether individual differences in alcohol use disorder symptom severity were associated with greater alcohol preference or the sensitivity of alcohol choice to opportunity and delay costs. The present study re-evaluated this concurrent choice design to determine whether alcohol use disorder symptom severity is associated with greater alcohol preference and/or greater discounting of opportunity and delay costs imposed on alcohol.

## Method

### Participants and questionnaires

One hundred and twenty-seven students who reported drinking at least occasionally (49% male) were recruited at the University of Exeter. Participants were aged between 18 and 51 (*M* = 21.4). At baseline, participants completed the Alcohol Use Disorders Identification Test (AUDIT) to index alcohol use disorder symptom severity (Babor et al. 2001) and the Timeline Follow Back (TLFB) questionnaire to index typical number of units of alcohol consumed per week (Sobell and Sobell 1992). AUDIT total scores were calculated by summing the ten items of that questionnaire, can range from 0 to 40 and are commonly split into the following categories: low-risk (0–7), hazardous (8–15), harmful (16–19) and possible dependent (20–40). The sample as a whole reported a mean AUDIT total score of 11.17 (*SD* = 6.03, range = 1–32), i.e. the mean was above the hazardous cutoff. Based on the AUDIT categories, there were 39 (31%) low-risk subjects, 57 (45%) hazardous subjects, 20 (16%) harmful subjects and 11 (9%) possible dependent subjects. The TLFB questionnaire indicated that the sample as a whole consumed an average of 14.17 units of alcohol per week (*SD* = 14.08, range = 0–75) estimated from the 2 weeks prior to testing. This average is right on the limit of 14 units per week proposed by the UK chief medical officers’ guidelines. Of the sample, 81 (64%) subjects drank less than this limit, and 46 (36%) drank more than this limit. There was a significant correlation between AUDIT total scores and average units per week estimated by the TLFB questionnaire, *r* = .69, *p* < .001. These findings suggest that the student sample contained a substantial proportion of drinkers above the hazardous cutoff (69%) and that the AUDIT total score was a valid estimate of alcohol use. Ethical approval was obtained from the University of Exeter Research Ethics Committee and subjects provided informed written consent.

### Concurrent choice task

**Fig. 1:**
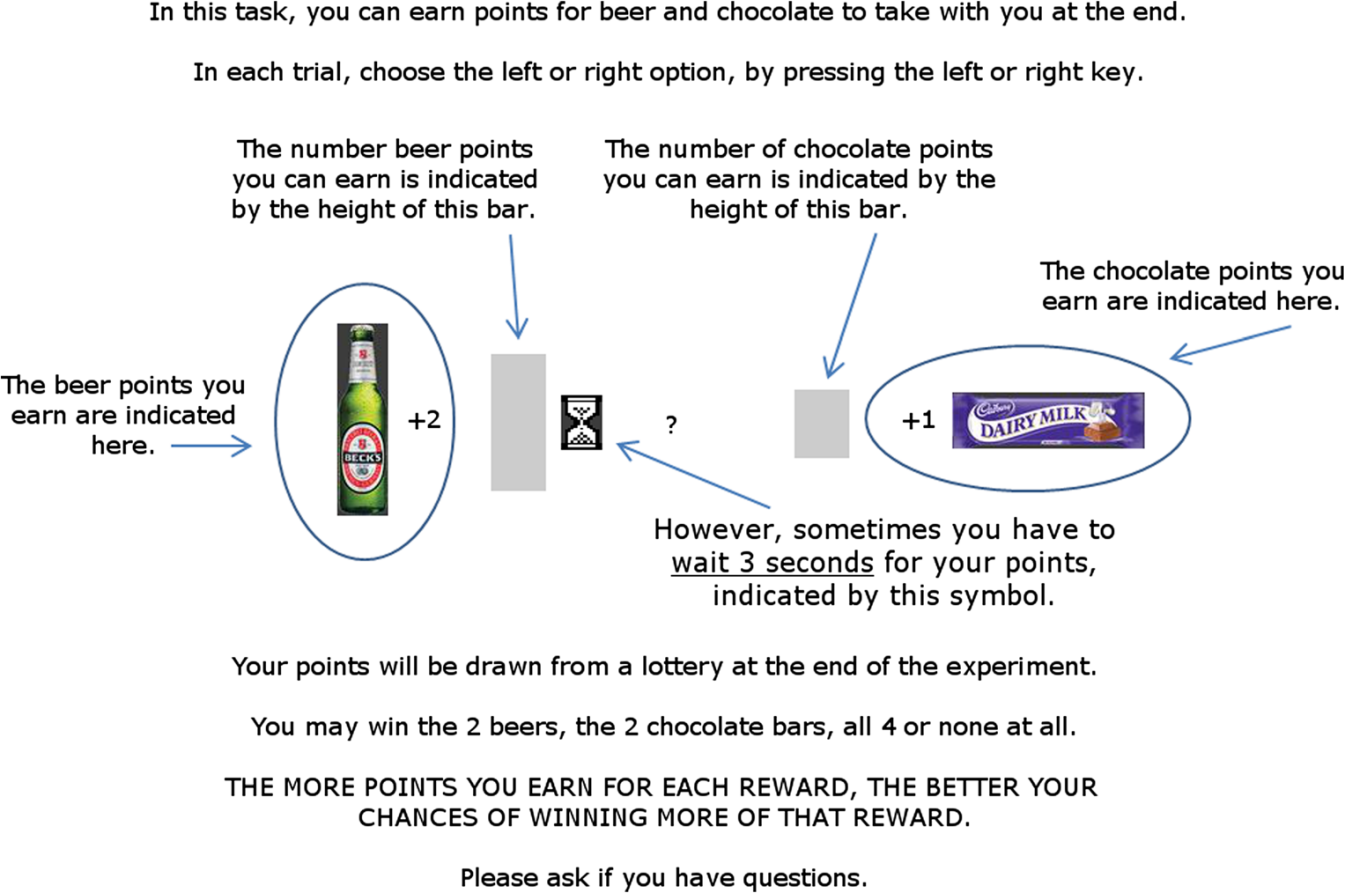
The instruction screen presented to participants at the start of the concurrent choice task. The left and right arrow keys were used to choose alcohol or chocolate points on offer (response-reward contingencies were counterbalanced between-subjects). The magnitude of the alcohol and chocolate points on offer was signalled by the height of the two grey bars. An hourglass symbol signalled whether a 3-s delay would be imposed on the receipt of the alcohol or chocolate reward, or neither. Following choice of the left or right option, a picture of the selected reward was displayed alongside the number of points earned for that reward (after a delay if this was imposed). Reward points were + 1, + 2 or + 3 signalled by the height of the grey bar. The relative magnitude of alcohol versus chocolate points was manipulated across five conditions (− 2, − 1, 0, + 1, + 2), and delay was manipulated across three conditions (delay alcohol, no delay, delay chocolate)

Figure 1 shows the on-screen instructions which informed participants about the nature of the task. Physical rewards were present on the desk between the screen and the keyboard: two 275 ml bottles of Becks beer and two 45 g bars of Dairy Milk chocolate. On-screen instructions stated that participants could earn points for the alcohol and chocolate rewards and that ‘points will be drawn from a lottery at the end of the experiment’. This statement was framed to give participants the impression that their response choices in the task had a direct impact on their chances of receiving the two rewards at the end. However, this instruction was a deception—all participants received a small chocolate bar at the end of testing irrespective of their choices.

For a random half of participants, the left key produced the alcohol reward and the right key produced the chocolate reward. These response-reward contingencies were reversed for the remaining half of participants. The position of rewards on the instructions page (Fig. 1) was congruous with the response-reward contingencies in the task. Participants completed 90 choice trials. At the start of each trial, participants were presented with two vertical grey bars in the left or right position which represented the magnitude of the alcohol and chocolate rewards on offer (small = 1, medium = 2 and large = 3 points). If an hourglass symbol was also present next to the bar, this indicated that a delay of 3 s would be imposed on receiving the reward (participants ultimately received the reward after the delay, so the cost of selecting the delayed choice was a lengthening of the study procedure by 3 s). Participants then made a choice between the left or right key response, and the reward was presented. If the alcohol choice was selected, a picture of a 275-ml bottle of Becks beer was presented, whereas if the chocolate choice was selected, a picture of a 45-g bar of Dairy Milk chocolate was presented. The picture of the selected reward was accompanied by a number, + 1, + 2 or + 3, which represented the number of points earned for that reward (corresponding to the height of the grey bar at the start of the trial). Finally, if the selected grey bar had an hourglass symbol next to it at the start of the trial, a 3-s delay was imposed between the choice of that option and the presentation of the reward picture and points (given that participants believed that the actual physical rewards—beer and chocolate—would be given to them at the end of the task, the delay to obtain the actual rewards imposed by choosing the delayed options was the sum of the 3 s delays).

There were 30 trials in which no delay was imposed on either reward (no hourglass symbol next to either grey bar). Across these 30 trials, there were five conditions that manipulated the magnitude of the alcohol and chocolate points on offer. Alcohol could be worth two fewer points than chocolate (1/3; six trials), 1 less point (1/2, 2/3; three trials each) equal points (1/1, 2/2, 3/3; two trials each), 1 more point (2/1, 3/2; three trials each) or 2 more points (3/1; six trials). These five conditions were coded as − 2, − 1, 0, + 1 and +2 respectively, reflecting the relative difference in the alcohol versus chocolate points on offer. There were 30 identical trials with the delay imposed on the alcohol choice and another 30 identical trials with the delay imposed on the chocolate choice. The 90 trials were selected at random without replacement. The dependent variable was percent choice of alcohol over chocolate in the five conditions that manipulated the relative magnitude of alcohol points (− 2, − 1, 0, + 1, + 2) and three conditions that manipulated delay to reward points (delay alcohol, no delay, delay chocolate).

## Results

### Effect of the relative magnitude of alcohol points on alcohol choice

**Fig. 2:**
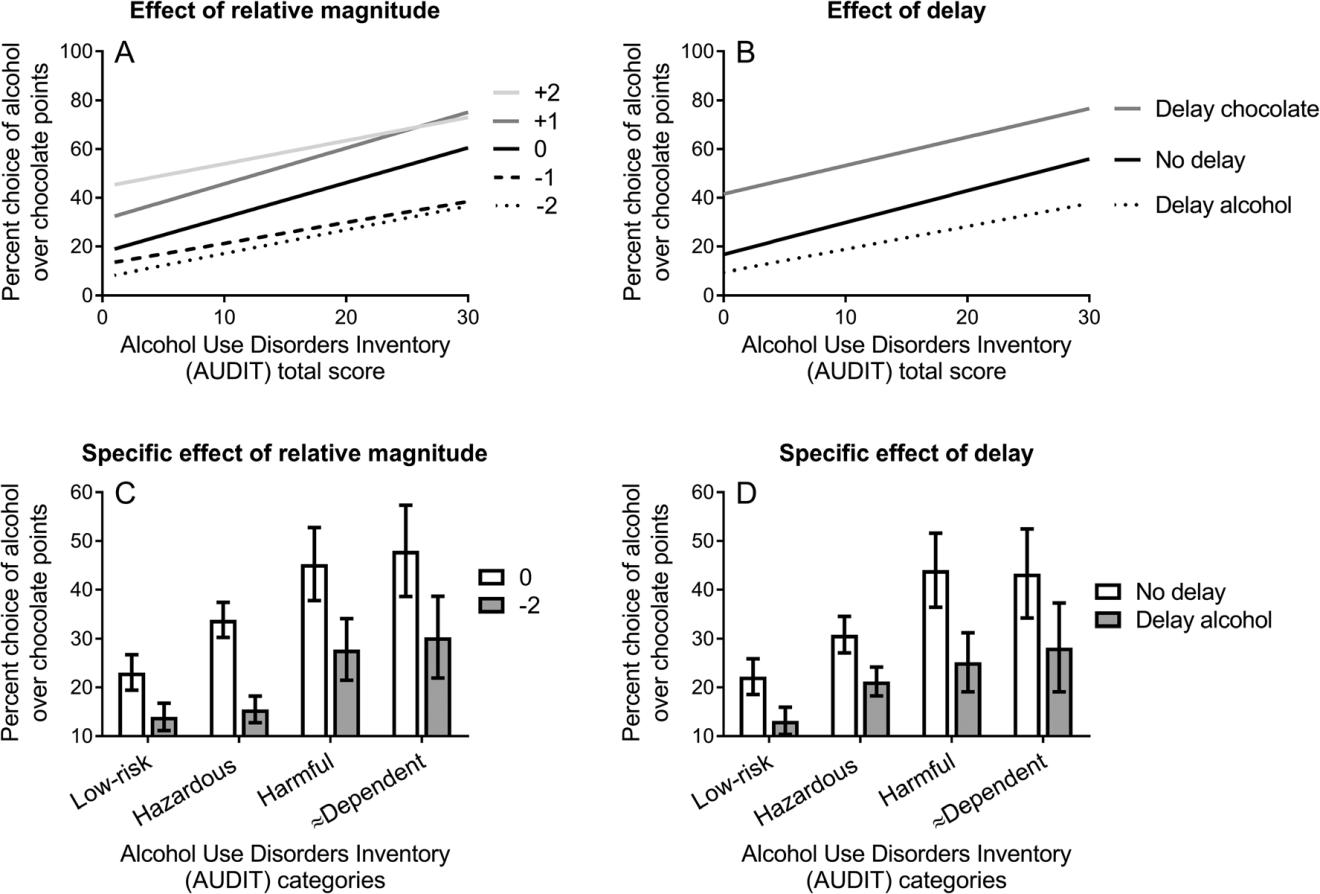
**a** The percent choice of alcohol over chocolate points in five conditions that manipulated the relative magnitude of the alcohol versus chocolate points (− 2, − 1, 0, + 1, + 2), as a function of alcohol use disorder symptom severity. **b** The percent choice of alcohol over chocolate points in three conditions that manipulated the delay imposed on receipt of these rewards (delay alcohol, no delay, delay chocolate), as a function of alcohol use disorder symptom severity. **c** The percent choice of alcohol over chocolate points in two conditions where alcohol and chocolate points were of equal magnitude (the 0 condition) and where alcohol was worth two fewer points than chocolate (the − 2 condition), to explore the extent to which opportunity costs (the possible loss of a valuable alternative) reduced alcohol choice. The sample was split into AUDIT categories reflecting alcohol dependence symptom scores, to better explore performance difference within each category: low-risk = scores 0–7; hazardous = scores 8–15; harmful = scores 16–19; and possible (≈) dependent = scores 20–40. **d** The percent choice of alcohol over chocolate points when no delay was imposed on rewards and when alcohol was delayed, to test the specific effect of delay costs on alcohol choice. The sample was split into AUDIT categories reflecting dependence symptom severity

Figure 2a shows the percent choice of alcohol over chocolate points in the five conditions that manipulated the relative magnitude of alcohol versus chocolate points (− 2, − 1, 0, + 1, + 2) as a function of AUDIT scores. A general linear model (GLM) was performed on these data, incorporating percent choice of alcohol over chocolate as the dependent variable, relative magnitude of alcohol points as the within-subjects variable and AUDIT total scores as a continuous predictor variable. There was a significant main effect of the relative magnitude of alcohol points on percent alcohol choice, *F*(4,500) = 20.79, *p* < .001, ηp^2^ = .143, indicating that alcohol choice tracked the relative magnitude of the alcohol points. As can be seen in Fig. 2a, percent alcohol choice increased with the relative magnitude of alcohol versus chocolate points offered in the five conditions: − 2 (*M* = 18.24, *SD* = 22.32), − 1 (*M* = 22.27, *SD* = 23.54), 0 (*M* = 33.55, *SD* = 28.22), + 1 (*M* = 47.42, *SD* = 32.23) and + 2 (*M* = 55.07, *SD* = 33.22). Within-subjects ANOVAs contrasting all possible pairs of the five relative magnitude conditions indicated all contrasts were significant, *F*s(1126) > 12.25, *p*s ≤ .001, ηp^2^s > .089.

In the overall GLM, there was also a main effect of AUDIT, *F*(1,125) = 11.75, *p* = .001, ηp^2^ = .086, indicating that alcohol use disorder symptom severity was associated with an increased preference for alcohol over chocolate, across conditions. The Pearson correlation between AUDIT scores and overall percent alcohol choice was *r* = .29, *p* = .001.

Finally and most importantly, in the overall GLM, there was no significant interaction between AUDIT scores and the relative magnitude of alcohol points, *F*(4,500) = 1.25, *p* = .289, ηp^2^ = .010. This finding indicates that as alcohol use disorder symptom severity increased, there was no difference in the sensitivity of alcohol choice to manipulation of the relative magnitude of alcohol points. Both the decrease in alcohol choice when alcohol was worth relatively less (the − 1 and − 2 conditions; i.e. impact of opportunity costs) and the increase in alcohol choice when alcohol was worth relatively more (+ 1 and + 2 conditions), compared to the 0 condition (where rewards were of equal magnitude), were comparable as a function of alcohol use disorder symptom severity. These findings suggest that alcohol use disorder symptoms are not associated with greater discounting of opportunity costs imposed on alcohol.

### Effect of delay on alcohol choice

Figure 2b shows the percent choice of alcohol over chocolate points in the three conditions of the delay manipulation (delay alcohol, no delay, delay chocolate), as a function of AUDIT scores. A GLM was performed on these data, incorporating percent choice of alcohol over chocolate as the dependent variable, delay condition as the within-subjects variable and AUDIT scores as a continuous predictor variable. There was a significant main effect of delay condition on percent alcohol choice, *F*(4,250) = 24.17, *p* < .001, ηp^2^ = .162, indicating that choice was modified by the delays imposed on rewards. As can be seen in Fig. 2b, percent alcohol choice was lowest when the delay was imposed on alcohol (*M* = 19.97, *SD* = 22.86), intermediate with no delay (*M* = 31.34, *SD* = 28.64) and the greatest when the delay was imposed on chocolate (*M* = 54.62, *SD* = 31.17). Within-subjects ANOVAs contrasting all possible pairs of the three delay conditions indicated that every contrast was significant, *F*s(1126) > 44.73, *p*s ≤ .001, ηp^2^s > .262.

In the overall GLM, there was also a main effect of AUDIT identical to the GLM that tested the relative magnitude of points, above. Finally, and most importantly, there was no significant interaction between AUDIT scores and delay condition, *F*(2,250) = 0.48, *p* = .622, ηp^2^ = .004. This finding indicated that as alcohol use disorder symptom severity increased, there was no difference in the sensitivity of alcohol choice to the delays imposed on alcohol and chocolate rewards. Both the decrease in alcohol choice when alcohol was delayed (i.e. the impact of delay costs) and the increase in alcohol choice when chocolate was delayed, relative to the no delay condition, were comparable as a function of alcohol use disorder symptom severity. These findings suggest that alcohol use disorder symptoms are not associated with greater discounting of delay costs imposed on alcohol.

### Specific contrasts to test a priori predictions

Specific contrasts were undertaken to test directly the prediction that alcohol use disorder symptoms are associated with greater discounting of opportunity and delay costs on alcohol choice. Figure 2c shows the percent choice of alcohol over chocolate in conditions where alcohol and chocolate points were of equal magnitude (the 0 condition) and where alcohol was worth two fewer points than chocolate (the − 2 condition). This comparison tests the effect of opportunity costs (the possible loss of a valuable alternative) on alcohol choice. The horizontal axis shows the sample split into AUDIT categories reflecting alcohol use disorder symptom severity, to better explore performance difference within each category. An ANOVA was performed on these data with percent alcohol choice as the dependent variable relative magnitude condition as the within-subjects factor (0, − 2) and AUDIT category as the between-subjects factor (4). There was a significant main effect of relative magnitude, *F*(1,123) = 40.01, *p* < .001, ηp^2^ = .245, and a significant main effect of AUDIT category, *F*(3,123) = 4.51, *p* = .005, ηp^2^ = .099, but no significant interaction between relative magnitude and AUDIT category, *F*(3,123) = 1.36, *p* = .258, ηp^2^ = .032. These findings confirm the conclusions of the primary analysis (in Fig. 2a) that increasing the relative magnitude of the alternative reward (opportunity costs) decreased alcohol choice and, crucially, that alcohol use disorder symptom severity was not associated with greater discounting of opportunity costs on alcohol choice.

Figure 2d shows the percent choice of alcohol over chocolate in conditions where no delay was imposed on rewards and when alcohol was delayed, to test the specific effect of delays costs on alcohol choice. ANOVA was performed on these data with percent alcohol choice as the dependent variable delay condition as the within-subjects factor (no delay, delay alcohol) and AUDIT category as the between-subjects factor (4). There was a significant main effect of delay condition, *F*(1,123) = 41.55, *p* < .001, ηp^2^ = .253, and a significant main effect of AUDIT category, *F*(3,123) = 3.14, *p* = .028, ηp^2^ = .071, but no significant interaction between delay condition and AUDIT category, *F*(3,123) = 1.53, *p* = .211, ηp^2^ = .036. These findings confirmed the conclusions of the primary analysis (in Fig. 2b) that imposing a delay on alcohol reduced alcohol choice and, crucially, that alcohol use disorder symptom severity was not associated with greater discounting of delay costs imposed on alcohol.

## Discussion

The current study found that alcohol use disorder symptom severity indexed by the AUDIT was associated with increased choice of alcohol over chocolate in a concurrent choice procedure. This finding replicates previous studies which have also found that alcohol use disorder symptoms are associated with preferential alcohol choice (Hardy and Hogarth 2017; Hardy et al. 2017; Hogarth et al. 2018) and accords with studies which have found that cocaine dependence symptoms are associated with preferential cocaine choice (Moeller et al. 2013; Moeller et al. 2009) and that tobacco dependence symptom severity is associated with preferential tobacco choice (Chase et al. 2013; Hogarth and Chase 2011). These findings provide powerful, converging support for the prediction of behavioural economic theory that drug dependence is driven by the ascription of greater relative value to drug rewards (Bickel et al. 2014; Hursh et al. 2005; MacKillop 2016). On this account, drug use might persist despite costs simply because drug value exceeds the costs (Heyman 2013).

The study also found that alcohol choice could be effectively modified by manipulating the relative magnitude of the competing alternative reward (chocolate) and by imposing delays upon the two rewards, suggesting drug choice is an economic decision based on the weighing of rewards and costs. These findings are consistent with previous concurrent choice studies which have demonstrated that alcohol choice can be lawfully modified by manipulating the magnitude and delay of the alternative money reward (Vuchinich and Tucker 1983; Vuchinich et al. 1987). Additionally, concurrent choice studies with drugs other than alcohol have also modified drug choice by manipulating the relative magnitude of the alternative natural reward (Bickel et al. 1995; Campbell and Carroll 2000; Carroll and Lac 1993; Carroll et al. 1989; Ginsburg and Lamb 2018; Hatsukami et al. 1994; Higgins et al. 1994, 1996; LeSage 2009; Nader and Woolverton 1991, 1992; Stevens Negus 2003) and by imposing a delay on either reward (Ito and Nakamura 1998; Woolverton and Anderson 2006). Precisely how the rewards and costs associated with two different reinforcers are commensurated to determine choice between them remains to be resolved (Rangel et al. 2008; Redish et al. 2008). Such knowledge will be crucial for developing future decision-based interventions.

The most important contribution of the current study was to demonstrate that alcohol use disorder symptoms severity was not associated with greater discounting of opportunity or delay costs imposed on alcohol choice. Specifically, the reduction in alcohol choice produced by either the increased value of chocolate points or delay imposed on alcohol reward did not show any statistical decline as a function of either continuous or categorical AUDIT scores. It is particularly salient that the 20 harmful and 11 possible dependent participants showed no evidence of reduced sensitivity to opportunity or delay costs compared to the 57 hazardous or 39 low-risk drinkers, in the analysis of categorical AUDIT groups. It is an empirical question as to whether the failure to detect cost insensitivity in more severe student drinkers would generalise to older drinkers with a clinical diagnosis of alcohol dependence. However, the current study does clearly suggest that hazardous campus drinking, which is a problem in its own right, is probably not driven by greater cost discounting, but rather, by greater relative value ascribed to alcohol.

The failure to demonstrate cost insensitivity with increasing AUDIT scores is at odds with four lines of evidence which suggest that dependence is linked to cost discounting. First, alcohol dependence symptoms are sometimes associated with higher breakpoints in demand tasks, suggesting dependence is associated with the discounting of price costs (MacKillop et al. 2015), and student drinkers with a family history of alcoholism are less sensitive to the effect of imagined next-day responsibilities on reducing alcohol demand (Murphy et al. 2014). Second, alcohol dependence symptoms are associated with a steeper delay discounting of rewards, which could theoretically extend to neglect of future costs associated with alcohol (Lim et al. 2017; MacKillop et al. 2011; Petry 2001; Vuchinich and Simpson 1998). Third, drug users show deficits in reversal learning which could be driven by insensitivity to punishment of the incorrect response during reversal (Ersche et al. 2008; Fortier et al. 2008; Reiter et al. 2016; Vanes et al. 2014). Finally, rats that are impulsive or have had extended access to the drug are less sensitive than control rats to the suppression of drug self-administration by contingent shock punishment, despite comparable baseline self-administration rates, suggesting equivalent drug valuation and selective discounting of costs (Belin et al. 2008; Economidou et al. 2009; Pelloux et al. 2007; Pelloux et al. 2015; Vanderschuren and Everitt 2004).

Several limitations of the current study might explain the failure to demonstrate greater cost discounting with alcohol use disorder symptoms and hence the inconsistency with previous evidence. First, our student subjects, despite being categorised as harmful or possibly dependent by their AUDIT scores, may not have acquired the same deficit in decision-making that drives persistent alcohol use in clinically diagnosed drinkers. This proposal could be tested straightforwardly by running clinically diagnosed drinkers on the current procedure to determine if they show greater cost discounting than matched non-dependent controls. Second, the costs imposed on alcohol (loss of chocolate points or 3 s delay) may not have been strong enough to reveal individual differences, such as those found with shock punishment in animals. This could be tested straightforwardly by using shock within the current paradigm. Third, our use of chocolate as the alternative reinforcer may have increased variance in the preferential choice measure due to individual differences in chocolate liking, thereby reducing sensitivity to individual differences in cost discounting. Future studies might negate this risk by utilising an alternative reinforcer for which there is more homogenous liking, such as money. Fourth, participants were deceived that they could earn alcohol and chocolate rewards contingent on their choices in the task. This deception could have been communicated between participants, which would increase variance in the preferential choice measure, thereby reducing sensitivity to individual differences in the cost discounting. Finally, our lab procedure may have failed to detect individual differences in cost discounting because the costs imposed were too specific and were not ecologically valid. For instance, alcohol dependence may be associated with discounting of real delayed costs such as negative educational, career, health or legal consequences, but because the 3 s delay manipulation did not adequately model this cost, we failed to detect differential sensitivity to cost discounting. By contrast, demand tasks measure hypothetical alcohol consumption under costs such as price (MacKillop et al. 2015) or imagined next-day responsibilities (Murphy et al. 2014), which may have greater ecological validity and therefore greater sensitivity to individual differences in cost discounting. Employing more ecologically valid costs within the current model, for example, by having participants pay for rewards, or by measuring alcohol choice under conditions of imaged next-day responsibilities, might reveal individual differences in cost discounting. Altogether, the limitations of the current model suggest that cost discounting could be found to play a role in dependence if different procedures or participants were studied.

Alternatively, if one accepted the current data and concluded that alcohol use disorder symptoms are not associated with greater cost discounting, then one would have to explain the apparent published evidence supporting this claim. Accordingly, the finding that at-risk drinkers have higher breakpoints (MacKillop et al. 2015) or reduced sensitivity to next-day responsibilities (Murphy et al. 2014) could reflect the greater relative value ascribed to alcohol compared to money or next-day responsibilities. Second, the steeper delay discounting of dependent drinkers might be a strategy developed through experience of unpredictable environments, rather than reflecting a constitutional neglect of future costs of alcohol. Third, drug users’ reversal deficits may stem from a general impairment (e.g. reduced prediction error coding, cognitive inflexibility, task disengagement), rather than a specific deficit in punishment sensitivity. Finally, insensitivity to the suppressive effects of shock on drug self-administration found in impulsive or extended drug access rats may not reflect cost discounting per se, but rather, may reflect greater value ascribed to the drug which was not effectively assessed by the single lever self-administration procedures used in previous studies (Bentzley et al. 2014; Pelloux et al. 2015). Altogether, this analysis and the current data weaken support for the claim that human drug dependence is driven by discounting costs associated with drug use. However, replication of the current effects with different participants and conditions is needed to substantiate this conclusion.

The current findings have clinical implications. The finding that alcohol choice is an economic decision based on weighing the rewards and costs of alcohol versus competing non-drug alternatives suggests that alcohol treatments should focus on (a) decreasing the value of alcohol, (b) increasing the costs of alcohol, (c) increasing the value of competing rewards and (d) decreasing the costs of competing rewards. There are many interventions which address these four decision variables including health education (Kleinot and Rogers 1982), taxation/minimum price policies (Chaloupka et al. 2002), contingency management (Higgins et al. 2004; Regier and Redish 2015), behavioural activation (Ross et al. 2016) and community-reinforcement (Meyers et al. 2011). The current study suggests that decision-oriented treatment research should focus on interventions that address all four decision variables simultaneously.
